# Functional analysis of PrkA - a putative serine protein kinase from *Mesorhizobium alhagi* CCNWXJ12-2 - in stress resistance

**DOI:** 10.1186/s12866-016-0849-6

**Published:** 2016-09-29

**Authors:** Xiaodong Liu, Yantao Luo, Zhefei Li, Gehong Wei

**Affiliations:** State Key Laboratory of Soil Erosion and Dryland Farming on the Loess Plateau, College of Life Sciences, Northwest A & F University, Yangling, Shaanxi 712100 China

**Keywords:** Serine protein kinase, Salt resistance, Antioxygenation, Na^+^ concentration measurement

## Abstract

**Background:**

Serine/threonine protein kinases are highly conserved kinases with a wide distribution in microbes and with multiple functions. *Mesorhizobium alhagi* CCNWXJ12-2, a α-proteobacterium which could be able to form symbiosis with *Alhagi sparsifolia* in northwest of China, contains a putative PrkA-family serine protein kinase, PrkA. In our previous study, the expression of *prkA* was found to be downregulated in high-salt conditions. To elucidate the function of *M. alhagi* PrkA, a *prkA* deletion mutant was constructed and the phenotypes of the mutant were analyzed.

**Results:**

The salt and alkaline tolerance and antioxidant capacity of the wild-type strain and the *prkA* deletion mutant was measured. Our results showed that the deletion mutant had higher salt and alkaline tolerance than the wild-type strain. The total cellular Na^+^ content was measured and showed no significant difference between the wild-type strain and the mutant. The *prkA* deletion mutant also showed a higher H_2_O_2_ tolerance than the wild-type strain. Therefore the activities of antioxidant enzymes were measured. Catalase activity was similar in the wild-type and the deletion mutant, while the superoxide dismutase activity in the mutant was higher than that in the wild-type.

**Conclusions:**

We firstly analyze the function of a serine protein kinase, PrkA, in *M. alhagi*. Our data indicate that PrkA could reduce the survival of *M. alhagi* under environmental stress and deletion of *prkA* dramatically improved the salt and alkaline tolerance and antioxidant capacity of *M. alhagi*.

## Background

Salinity and desiccation are major problems facing agriculture worldwide [1, 2]. *Mesorhizobium alhagi* CCNWXJ12-2 is a highly salt-tolerant and alkali-tolerant rhizobium which can form nodules with the desert plant *Alhagi sparsifolia* [3]. The nitrogen-fixing symbiosis formed between rhizobia and legumes can decrease the damage to plants caused by soil salinity; thus, there are an increasing number of studies on salt-tolerant rhizobia and their mechanism(s) of salt resistance [4, 5]. To determine the mechanism of salt resistance in *M. alhagi*, a global transcriptome comparison of *M. alhagi* was conducted in salt-free (no added salt) and high-salt conditions, and a downregulated putative serine kinase gene, *prkA*, was identified in high-salt [6].

PrkA is a highly conserved serine protein kinase with a wide distribution in bacteria and archaea [7]*.* The serine/threonine protein kinases play diverse roles in bacterial signal transduction and regulation by phosphorylating multiple substrates [8]. In the model bacterium *Escherichia coli*, the *prkA* homolog *yeaG* was regulated by leucine-responsive regulatory protein and may have a role in metabolic reprogramming to survive acidic and osmotic stress [9]. Recent reports showed that *yeaG* is also involved in adaptation to nutrient limitation [10, 11]. However, subsequent research showed that a *yeaG* deletion mutant showed no significant difference in salt tolerance and pH adaptation compared with the wild-type strain [12].

In *Mycobacterium tuberculosis*, the deletion mutant of *pknE* (a gene encoding a serine/threonine protein kinase) showed defective growth in conditions of neutral pH and on exposure to lysozyme, but a higher tolerance to acidic stress, sodium dodecyl sulfate (SDS), and kanamycin [13]. Proteomic and phosphoproteomic analysis of the *pknE* mutant of *M. tuberculosis* showed that PknE was involved in metabolism, dormancy, and suppression of some sigma factors and other kinases, and thus could play an important role in adaptive responses to hostile environments [14].

In *Bacillus subtilis*, PrkA was proved to be an inner spore coat protein and involved in spore formation, which is controlled by sigma factors [15, 16]. In *Streptococcus mutans*, the serine/threonine protein kinase annotated *pknB* was shown to play a significant role in biofilm formation, genetic competence, and acid resistance [17]. In *Rhizobium etli*, the expression of *prkA* was highly dependent on alarmones, guanosine, tetraphosphate, and guanosine pentaphosphate, but the Δ*prkA* mutant of *R. etli* showed no clear difference compared with the wild-type under osmotic, oxidative and heat stresses [7].

In this study, we constructed a Δ*prkA* (the *prkA* deletion mutant of *M. alhagi*) mutant and undertook a phenotypical analysis to understand the function of PrkA. Our results showed that the Δ*prkA* mutant grew better under high-salt (0.4 M NaCl) and alkaline (pH 9) conditions than the wild-type strain, and the survival rate of the mutant under oxidative stress was also higher than that of the wild-type. The total cellular Na^+^ content in the mutant was almost the same as that in the wild-type in high-salt conditions. Although the catalase (CAT) activity was similar in the wild-type and Δ*prkA* mutant, the superoxide dismutase (SOD) activity in Δ*prkA* mutant was higher than that in the wild-type. These results indicate that *prkA* could reduce the ability of *M. alhagi* in stress adaptation.

## Results and discussions

### Increased NaCl and alkali tolerance of mutant Δ*prkA*

There is only one annotated PrkA family serine protein kinase in the genome of *M. alhagi*, denoted PrkA [18]. We have previously found that the expression of *prkA* was downregulated in high-salt conditions through RNA-Seq validated by RT-qPCR [6]. To determine the function of *M. alhagi* CCNWXJ12-2 *prkA*, we constructed a deletion mutant and its tolerance to NaCl and alkali were tested.

**Fig. 1:**
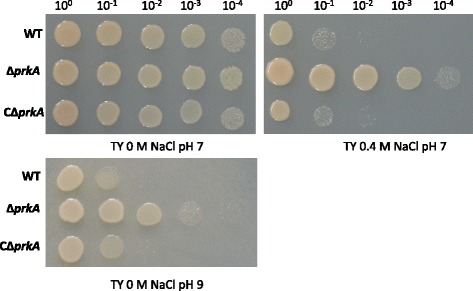
Sensitivity of the wild-type and mutant strains to NaCl and alkaline. Wild-type (WT), Δ*prkA* (*prkA* deletion mutant), and CΔ*prkA* (complement of Δ*prkA*) bacterial cells were grown to OD_600_ ≈ 0.8 in TY broth medium, adjusted to OD_600_ ≈ 0.2, and then serially diluted and spotted onto TY agar plates containing no additional NaCl or 0.4 M NaCl at neutral pH, and TY agar plates at alkaline pH (pH 9). Ten-fold serial dilutions are shown. The plates with no additional NaCl were incubated at 28 °C for 3 days, while the plates with 0.4 M NaCl and the plates at pH 9 were incubated for 5 days at the same temperature

The mutant Δ*prkA* had better salt and alkali resistance than the wild-type strain, while the complemented strain CΔ*prkA* had similar salt and alkali resistance to the wild-type strain (Fig. 1). The expression of *prkA* in the complementation strain was confirmed using reverse transcription PCR (data not shown).

In *Mycobacterium tuberculosis*, the *pknE* deletion mutant showed higher tolerance to acidic stress, SDS, and kanamycin than the wild-type, which means *pknE* is involved in stress adaptation [13]. Although the protein sequence similarity of *Mesorhizobium alhagi* PrkA and *Mycobacterium tuberculosis* PknE is only 12.9 %, the function of *pknE* in *M. tuberculosis* was to some degree similar to that of *prkA* in *M. alhagi* in stress adaption. However, our results showed that wild-type *M. alhagi* had the same resistance to antibiotics (kanamycin, gentamicin, ampicillin, tetracycline, streptomycin, and rifampicin; 50 ug/ml for all), SDS (0.01 %), and acid (pH 6) as did the mutant Δ*prkA* (data not shown).

In *E. coli*, the deletion mutant of *yeaG* showed no difference in salt tolerance compared with the wild-type [12], although the similarity in protein sequence between *E. coli* YeaG and *M. alhagi* PrkA is relatively high (65.5 %). Despite the similar genomic background of *prkA* in *R. etli* and *Mesorhizobium alhagi* (data not shown) and the high similarity in protein sequences (71.49 %), the *prkA* deletion mutant of *R. etli* showed no phenotype changes compared with the wild-type strain [7], in contrast to *M. alhagi* in the present work.

The functions of these genes in the different bacteria are obviously different. These results show that serine protein kinases can have very different functions in different bacteria.

### Measurement of total cellular Na^+^ content

**Fig. 2:**
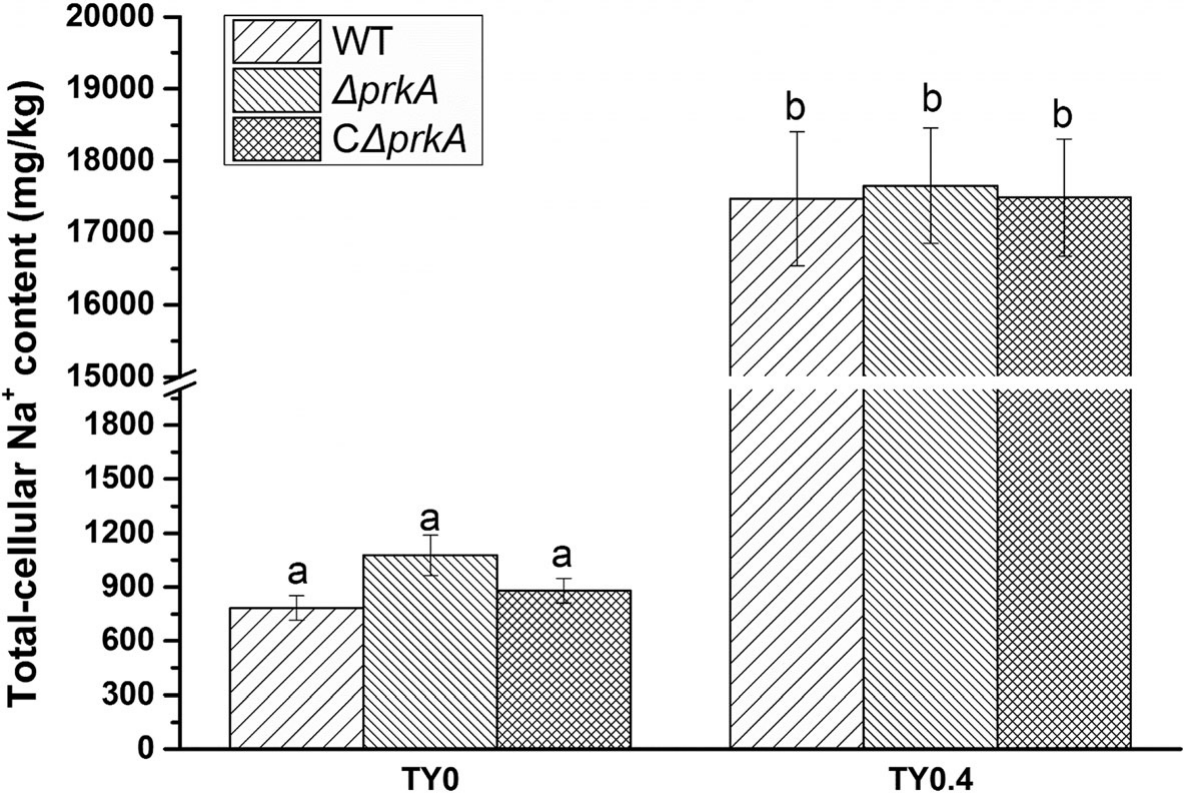
Measurement of total cellular Na^+^ concentration. Wild-type (WT), Δ*prkA* (*prkA* deletion mutant), and CΔ*prkA* (complement of Δ*prkA*) bacterial cells were grown to OD_600_ ≈ 0.8 in TY broth medium and then adjusted to OD_600_ ≈ 0.2. The diluted inocula were plated on TY agar plates with and without 0.4 M NaCl and inoculated at 28 °C for 5 days. Total cellular Na^+^ concentrations were measured using an atomic absorption spectrophotometer. The results are shown as the means of three biological replicates and the error bars indicate standard deviations. The significance of differences is shown at the *P* < 0.05 level (*t*-test). Different lowercase letters mean significant difference between two columns

Bacteria can efflux the extra Na^+^ from the cells by Na^+^/H^+^ antiporters using energy of proton motive force [19]. We have previously found that the expression of a Na^+^/H^+^ antiporter gene, *nhaA*, was upregulated in high-salt conditions [6]. Here we measured the total cellular Na^+^ content of the wild-type, Δ*prkA*, and CΔ*prkA* strains in salt-free and high-salt (0.4 M NaCl) conditions to find out whether PrkA influences the total cellular Na^+^ content. However, our results showed no significant difference among these three strains (*p* ≥ 0.05) when grown in the same conditions (Fig. 2). The Na^+^ content of the three strains grown on 0.4 M NaCl TY agar plates was almost 20-fold higher than that in the controls grown on salt-free medium. The similar Na^+^ content of the three strains implied that *prkA* does not depress the strain growth in high-salt conditions by adjusting the cellular Na^+^ content. Therefore, we speculate that the mechanism of PrkA depressing the growth of *Mesorhizobium alhagi* under salt stress is complex, which involves many components of metabolism.

### Increased antioxidative capacity of mutant *ΔprkA*

**Fig. 3:**
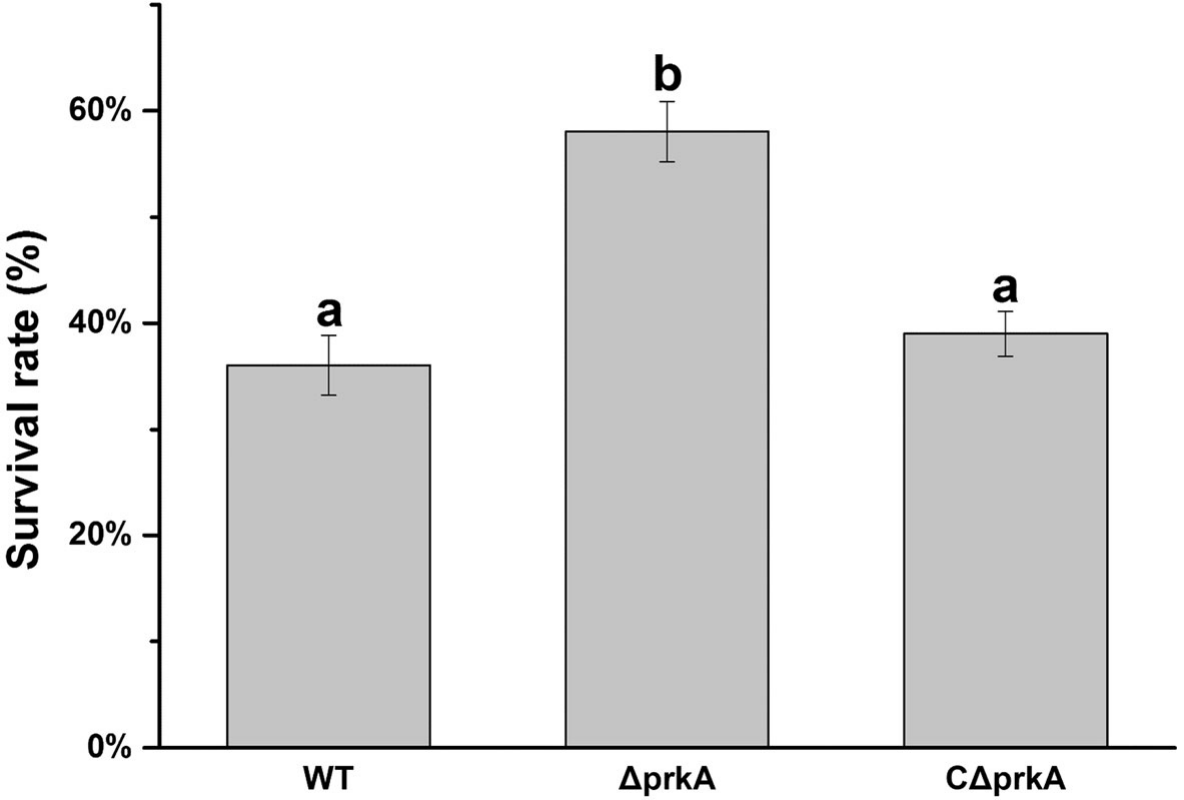
Survival rate of *M. alhagi* after H_2_O_2_ treatment. Wild-type (WT), Δ*prkA* (*prkA* deletion mutant), and CΔ*prkA* (complement of Δ*prkA*) bacterial cells were grown to OD_600_ ≈ 0.8 in TY broth medium, adjusted to OD_600_ ≈ 0.1, and then treated with 10 mM H_2_O_2_ for 30 min or incubated without H_2_O_2_ in otherwise identical conditions as a control. The percentage survival rate of the three stains was calculated as follows: [(CFU per ml after treatment with H_2_O_2_)/(CFU per ml before treatment with H_2_O_2_)] × 100. Data shown are the means of three independent experiments and the error bars indicate standard deviations. The significance of differences is shown at the *P* < 0.05 level (*t*-test). Different lowercase letters mean significant difference between two columns

Stressful conditions such as heat, acid and high-salt concentrations, can lead to secondary oxidative stress in bacteria [20]. Previous study has shown the intracellular level of reactive oxygen species (ROS) was increased significantly when cells were stressed by high salinity [21, 22]. Because high salinity can trigger a high level of intracellular ROS, we tested the oxidative resistance of the *M. alhagi prkA* deletion mutant to identify any antioxidant function of PrkA. The survival rates of the wild-type, Δ*prkA* and CΔ*prkA* strains treated with 10 mM H_2_O_2_ for 30 min showed a significant difference (Fig. 3); survival of Δ*prkA* treated with H_2_O_2_ was significantly (*p* ≤ 0.05) higher than that of the wild-type and complemented strains. These results suggest that PrkA depresses the antioxidative capacity of *M. alhagi*.

Although H_2_O_2_ can be damaging for rhizobium, it appears that H_2_O_2_ is required for successful infection. The overexpression of the housekeeping catalase in *Sinorhizobium meliloti* Rm*kat*B^++^ results in a delay of symbiosis formation and has negative effects on the development of infection threads [23].

Unfortunately, the symbiosis formation of *M. alhagi* and *Alhagi sparsifolia* is very unstable. Great effort has been made to conduct the plant experiments, but the results are always unreliable and unauthentic (data not shown). Therefore, we can only hypothesize that PrkA has positive effects on symbiosis formation.

### Antioxidant enzyme activity determination

**Fig. 4:**
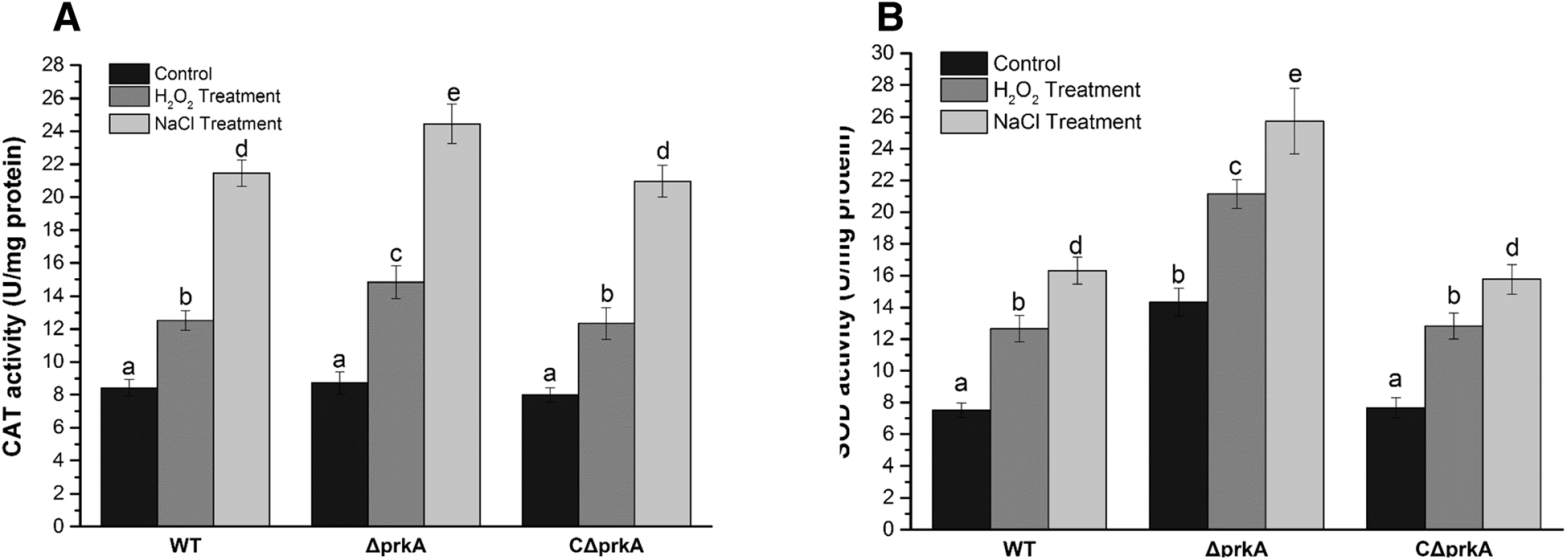
Measurement of antioxidant enzyme activities. Measurement of catalase (CAT, (**a**)) and superoxide (SOD, (**b**)) activities in wild-type *M. alhagi* (WT), Δ*prkA* (*prkA* deletion mutant), and CΔ*prkA* (complement of Δ*prkA*). Three independent biological experiments were conducted to measure the CAT and SOD activities. The error bars indicate standard deviations. The significance of differences is shown at the *P* < 0.05 level (*t*-test). Different lowercase letters mean significant difference between two columns

Catalase (CAT), superoxide dismutase (SOD) and peroxidase (POD) are major antioxidases in bacteria, which can eliminate the intracellular ROS [24]. Therefore, we measured the CAT, SOD, and POD activities of *M. alhagi* in different conditions. Figure 4 shows the enzyme activity in the three strains (without treatment, H_2_O_2_ treated, or 0.4 M NaCl treated). The CAT and SOD activities could be detected in all strains in each condition, while the POD activity was not detectable. We checked the RNA-Seq data and found that the genes coding CATs and SODs are highly expressed in *M. alhagi*, while PODs are only expressed at a low level [6]; thus POD activity in cells may have been below the detection limit of the kit used in our experiments.

In control cells (normal conditions), the CAT activities of the three strains were almost the same, while the SOD activity of Δ*prkA* was significantly higher (*p* ≤ 0.05) than that in the other two strains. Salt stress and H_2_O_2_ treatment triggered increased CAT and SOD activities in all three strains (Fig. 4). The CAT and SOD activities under high-salt stress were markedly higher than those in the H_2_O_2_ treatment group, which could also suggest that high-salt stresses trigger oxidative stress. The lower CAT and SOD activities in the H_2_O_2_ treatment group were possibly caused by the low H_2_O_2_ concentration or the short treatment time.

The CAT and SOD activities of Δ*prkA* under salt stress and H_2_O_2_ treatment were extremely significantly higher (*p* ≤ 0.001) than those of the wild-type and CΔ*prkA* (Fig. 4). The increase in CAT activity units of Δ*prkA* compared to the other two strains was much smaller than that in SOD activity units. Therefore, we hypothesize that PrkA influences the antioxidative capacity of *M. alhagi* mainly by affecting SOD activity. However, in favorable conditions (e.g., as in the control group), the high expression of SOD genes in Δ*prkA* could waste energy. PrkA may play a role in control of SOD gene expression. The adjustment of SOD gene expression may need the high expression of *prkA* in salt-free conditions.

## Conclusions

A *prkA* deletion mutant has been constructed to study the function of PrkA in *M. alhagi*. The data suggest that the ability to survive some abiotic stresses was increased by knocking out *prkA*. Moreover, we showed similarities and differences in the functions of PrkA and its homologs in *R. etli* and other bacteria. Our results suggest that it is possible to increase the salt and alkali tolerance of a bacterium by constructing specific mutants in genes highlighted by RNA-Seq data. To our knowledge, this is the first report to identify the function of PrkA in stress adaption of *Mesorhizobium* and to construct an increased salt-tolerant rhizobial mutant. Most curious and interesting was that the high expression of *prkA* made *M. alhagi* more vulnerable to high salinity. Because of the unstable symbiosis formation in plant tests, the role *prkA* plays in symbiosis formation is unclear and needs more efforts to be illuminated.

## Methods

### Bacterial strains and growth conditions

Table 1 lists bacterial strains and plasmids used in this study. The purified bacteria were typically grown in tryptone-yeast extract (TY) broth (5 g tryptone, 3 g yeast extract, and 0.7 g CaCl_2_ · 2H_2_O per liter) at 28 °C for *M. alhagi* and its mutants, or Luria-Bertani broth (10 g tryptone, 5 g yeast extract, and 10 g NaCl per liter) at 37 °C for *Esherichia coli*. SM agar plates (10 g mannitol, 0.5 g K_2_HPO_4_, 0.5 g KNO_3_, 0.2 g MgSO_4_ · 7H_2_O, 0.1 g CaCl_2_, 0.1 g NaCl, and 15 g agar per liter) was used to isolate the mutants. All bacteria were incubated in aerobic conditions. Where necessary, antibiotics were added as the following concentrations: kanamycin, 100 μg/ml; gentamicin 50 μg/ml.

**Table 1:** Bacterial strains and plasmids used in this study

Strain or plasmid	Description^a^	Source or reference
Escherichia coli		
DH5α	*endA hsdR17 supE44 thi-1 recA1 gyrA relA1* Δ(*lacZYA-argF*)*U169 deoR* [*Φ80 dlacΔ(lacZ)M15]*	[27]
S17-1λ*pir*	Tp^r^ Str^r^ *recA thi pro hsdR hsdM* ^+^ RP4::2-Tc::Mu::Km Tn7 λpir lysogen	[28]
DH-PrkA	DH5α carrying pK18prkA	This study
DH-CPrkA	DH5α carrying pBLprkA	This study
Mesorhizobium alhagi		
XJ12-2	Wild-type	[18]
*ΔprkA*	XJ12-2 *ΔprkA*	This study
C*ΔprkA*	*ΔprkA* carrying pBLprkA	This study
Plasmids		
pK18*mobsacB*	Suicide vector derived from plasmid pK18; Mob^+^ *sacB* Km^r^	[25]
pBBR1MCS-5	Broad-host-range cloning vector; Gm^r^	[29]
pK18prkA	pK18*mobsacB*::*prkA*	This study
pBLprkA	pBL carrying *prkA*	This study

^a^Km^r^, kanamycin resistance; Gm^r^, gentamicin resistance

### Plasmid construction

The primers used to construct the *prkA* deletion mutant and the complementation strain are listed in Table 2. Primers PrkA-US and PrkA-UA were used to amplify a 208-bp upstream fragment of *prkA* with an *Eco*RI restriction enzyme site at the 5′ end and a *Bam*HI site at the 3′ end. Primers PrkA-DS and PrkA-DA were used to amplify a 444-bp downstream fragment of *prkA* containing a *Bam*HI restriction enzyme site at the 5′ end and an *Xba*I site at the 3′ end. The two fragments were then digested with the relevant restriction enzymes using standard protocols. The two digested fragments were then cloned into plasmid pk18*mobsacB* digested with the same enzymes to generate plasmid pK18prkA, which was verified by sequencing [25].

**Table 2:** Primers used for mutant and complement strain construction

Primer	Sequence^a^ (5′-3′)
PrkA-US	CGGAATTCTCCTTCGTTCGCGGA
PrkA-UA	CGGGATCCGACCTCGTTGCCGA
PrkA-DS	CGGGATCCGCCTTCCCATATCAG
PrkA-DA	GCTCTAGACACTTCCTCCGTGTCGT
CPA	ATGAAGTCGCTTAATCCCCCGGGCATCGGGTCCGGCGCCGGATCGGG
CPB	TAACAAAATATTAACGCCCCGGGTCAGCCCGCCTTGTTGACGCGCATG

^a^Restriction enzyme sites are underlined

To construct the plasmid for *prkA*-complementation, a DNA fragment containing full-length *prkA* and a putative promoter of *prkA* (500 bp upstream of *prkA*) was amplified from genome DNA of *M. alhagi* using primers CPA and CPB. The PCR product was then purified using a Universal DNA Purification Kit (Tiangen, China). The purified PCR product was cloned into plasmid pBBR1MCS-5 digested with *Sma*I, using the ClonExpress MultiS One Step Cloning Kit (Vazyme Biotech, China), to generate the complementation plasmid pBLprkA, which was verified by sequencing.

### Mutant and complement construction

To construct the *prkA* deletion mutant, we first transformed pK18prkA into *E. coli* strain S17-1λ*pir*. Then a biparental mating procedure was used, as described previously [26], to transform pK18prkA from *E. coli* S17-1λ*pir* into *M. alhagi* CCNWXJ12-2. Briefly, cultures of *E. coli* S17-1λ*pir* (OD_600_ [optical density at 600 nm] ≈ 0.6) and *M. alhagi* CCNWXJ12-2 (OD_600_ ≈ 0.8) were mixed together in the ratio 1:2 (v/v) and cultured on a TY agar plate for 3 days. Single exchange (plasmid pK18prkA integrated into genome DNA of *M. alhagi*) cells of *M. alhagi* were selected using SM agar plates containing kanamycin. Double exchange mutants (*prkA* deletion mutant) were then isolated using TY agar plates containing sucrose (5 g/100 ml). Both of single-exchange and double-exchange mutants were verified by colony PCR and sequencing.

For genetic complementation, pBLprkA was transformed from *E. coli* S17-1λ*pir* into *M. alhagi* CCNWXJ12-2 by biparental mating. SM agar plates containing gentamicin were used to isolate the complementation mutant, and colony PCR and sequencing were used to verify the mutant strain.

### Salt and alkali resistance assays

Wild-type *M. alhagi*, Δ*prkA*, and CΔ*prkA* were first grown in 20 ml TY broth to OD_600_ ≈ 0.8. Then suspensions (20 μl) of the three cultures were added to 20 ml TY broth and grown to OD_600_ ≈ 0.8. The inocula were adjusted to OD_600_ ≈ 0.2 with sterile water and then serially diluted to 10-fold. Then 5 μl of each diluted inoculum were respectively spotted onto TY agar plates at pH 7 containing no NaCl (control) or 0.4 M NaCl (salt treatment), or TY agar plates at pH 9 containing no NaCl (alkaline treatment). The pH of TY agar medium was adjusted using NaOH solution (1 M) before sterilization.

### Measurement of total cellular Na^+^ content

Inocula were prepared as described in the section on the salt and alkali resistance assay. Five hundred microliters of each inoculum (OD_600_ ≈ 0.2) were plated on TY agar plates with and without 0.4 M NaCl and inoculated at 28 °C for 7 days. The bacteria were collected in 1.5-ml sterile tubes and dried using an air dryer at 50 °C for 12 h. The total cellular Na^+^ content was measured using an atomic absorption spectrophotometer (Hitachi, Tokyo, Japan).

### Sensitivity assay of H_2_O_2_

Inocula (OD_600_ ≈ 0.1) were treated with or without 10 mM H_2_O_2_ for 30 min, then serially diluted and plated on TY agar plates. The colonies were counted after 10 days of growth at 28 °C. The percentage survival rate of the three stains was calculated as follows: [(CFU per ml after treatment with H_2_O_2_)/(CFU per ml before treatment with H_2_O_2_)] × 100.

### Antioxidant enzyme activity assays

Wild-type *M. alhagi*, Δ*prkA* and CΔ*prkA* were grown in TY broth medium to OD_600_ ≈ 0.8. Each strain (20 μl suspension) was added to two bottles of 20 ml TY broth medium without additional NaCl and one bottle of 20 ml TY broth medium containing 0.4 M NaCl. Then the bacteria were grown to OD_600_ ≈ 0.8. One bottle of TY broth medium without NaCl was set as the control group and the other was treated with 0.1 mM H_2_O_2_ for 30 min (H_2_O_2_ treatment). Then all groups for the three strains were collected in 50-ml tubes by centrifugation at 8000 g for 5 min. The cells were lysed by ultrasonication. The enzyme activities of CAT, SOD and POD were determined using a CAT Assay Kit, a SOD Assay Kit, and a POD Assay Kit (Suzhou Comin Biotechnology, China), respectively, according to the manufacturer’s protocols. Total soluble protein concentration was measured using a BCA Protein Assay Kit (CWBIO, China).

### Statistical analysis

Statistical differences between the control and treatment groups of different strains were assessed by *t*-test using SPSS software version 15 (SPSS Inc., Chicago, IL, USA). Differences were considered to be significant at a probability level of P <0.05.

## Abbreviation

CAT: Catalase
CFU: Colony forming units
CΔ*prkA*: The complemented strain of Δ*prkA*
POD: Peroxidase
SDS: Sodium dodecyl sulfate
SOD: Superoxide dismutase
Δ*prkA*: The *prkA* deletion mutant of *Mesorhizobium alhagi* CCNWXJ12-2
